# Direct Immunochemiluminescent Assay for proBNP and Total BNP in Human Plasma proBNP and Total BNP Levels in Normal and Heart Failure

**DOI:** 10.1371/journal.pone.0053233

**Published:** 2013-01-24

**Authors:** Toshio Nishikimi, Hiroyuki Okamoto, Masahiro Nakamura, Naoko Ogawa, Kazukiyo Horii, Kiyoshi Nagata, Yasuaki Nakagawa, Hideyuki Kinoshita, Chinatsu Yamada, Kazuhiro Nakao, Takeya Minami, Yoshihiro Kuwabara, Koichiro Kuwahara, Izuru Masuda, Kenji Kangawa, Naoto Minamino, Kazuwa Nakao

**Affiliations:** 1 Department of Medicine and Clinical Science, Kyoto University Graduate School of Medicine, Kyoto, Japan; 2 Diagnostics Division, Shionogi & Co., Ltd, Osaka, Japan; 3 Takeda Hospital Medial Examination Center, Kyoto, Japan; 4 Department of Biochemistry National Cerebral and Cardiovascular Center Research Institute, Osaka, Japan; 5 Department of Molecular Pharmacology, National Cerebral and Cardiovascular Center Research Institute, Osaka, Japan; University of Buenos Aires, Cardiovascular Pathophysiology Institute, Argentina

## Abstract

**Background:**

Recent studies have shown that in addition to brain (or B-type) natriuretic peptide (BNP) and the N-terminal proBNP fragment, levels of intact proBNP are also increased in heart failure. Moreover, present BNP immunoassays also measure proBNP, as the anti-BNP antibody cross-reacts with proBNP. It is important to know the exact levels of proBNP in heart failure, because elevation of the low-activity proBNP may be associated with the development of heart failure.

**Methodology/Principal Findings:**

We therefore established a two-step immunochemiluminescent assay for total BNP (BNP+proBNP) and proBNP using monoclonal antibodies and glycosylated proBNP as a standard. The assay enables measurement of plasma total BNP and proBNP within only 7 h, without prior extraction of the plasma. The detection limit was 0.4 pmol/L for a 50-µl plasma sample. Within-run CVs ranged from 5.2%–8.0% in proBNP assay and from 7.0%–8.4% in total BNP assay, and between-run CVs ranged from 5.3–7.4% in proBNP assay and from 2.9%–9.5% in total BNP assay, respectively. The dilution curves for plasma samples showed good linearity (correlation coefficients = 0.998–1.00), and analytical recovery was 90–101%. The mean total BNP and proBNP in plasma from 116 healthy subjects were 1.4±1.2 pM and 1.0±0.7 pM, respectively, and were 80±129 pM and 42±70 pM in 32 heart failure patients. Plasma proBNP levels significantly correlate with age in normal subjects.

**Conclusions/Significance:**

Our immunochemiluminescent assay is sufficiently rapid and precise for routine determination of total BNP and proBNP in human plasma.

## Introduction

Brain (also known as B-type) natriuretic peptide (BNP) has been used as a biomarker of heart failure for more than a decade [1]. Indeed, guidelines for the treatment of heart failure recommend measurement BNP before making a diagnosis [2], [3]. During the process by which BNP is secreted from cardiac myocytes, its 108-amino acid precursor, proBNP, is cleaved to form the 32-amino acid peptide BNP and the 76-amino acid peptide N-terminal proBNP fragment (NT-proBNP) [4]. Recent studies have shown that in addition to BNP and the NT-proBNP, levels of uncleaved proBNP are also considerably increased in plasma of patients with heart failure [5], [6], [7]. This is noteworthy in part because the immunoassay system currently being used to measure BNP levels also detects proBNP, as the anti-BNP antibody cross-reacts with proBNP. Consequently, the present assay system actually measures not the active BNP level, but the total BNP (BNP+proBNP) level [8].

It is important to know the proBNP level and/or proBNP/total BNP ratio in heart failure, because proBNP has much less ability to induce cGMP production (about 13–17%) than BNP, and higher levels of the low-activity proBNP may be associated with the development of heart failure [7]. Consistent with that idea, we recently used the combination of gel-filtration and a fluorescent immunoenzyme assay with BNP extracted from plasma to show that although proBNP/total BNP ratios vary widely in heart failure, they are higher in cases of ventricular overload than in atrial overload [6]. Unfortunately, the method used in that study requires a great deal of time and effort, and extraction of the peptide from plasma may cause underestimation of the proBNP levels due to its high adsorptive property [9].

**Figure 1:**
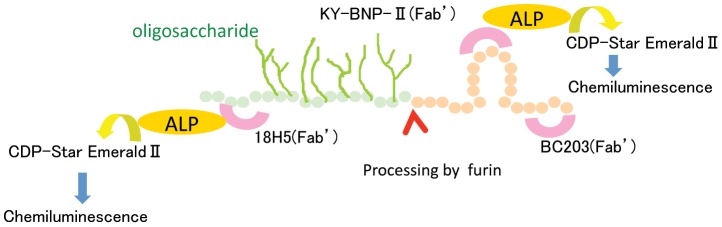
Schematic diagram of the total BNP and proBNP assay systems. BC203(Fab') is a common capture antibody in both systems. KY-BNP-II(Fab') is the detection antibody for the total BNP assay, and 18H5(Fab') is the detection antibody for the proBNP assay. ALP: Alkaline phosphatase; CDP-Star EmeraldII (Chemiluminescent Substrate): Disodium 2-chloro-5-(4-methoxy-spiro{1,2-dioxetane-3,2′-(5′-chloro)-tricyclo [3,3,1,13,7]decan}-4-yl)-1-phenyl phosphate.

To overcome those shortcomings, we developed a sensitive method to more quickly and easily measure levels of proBNP and total BNP. Our idea was to make a sandwich immunoassay using a common capture antibody recognizing the C-terminal region of both BNP and proBNP and detection antibodies that recognize different epitopes: the N-terminal region of proBNP and the ring structure of BNP (Figure 1). Using this approach, we were able to develop a sensitive immunochemiluminescent assay for proBNP and total BNP in plasma. Here, we report on the assay's performance and its use to compare plasma levels of total BNP and proBNP in healthy subjects and patients with heart failure. In addition, we measured NT-proBNP and compared it with total BNP and proBNP.

## Materials and Methods

All patients provided written informed consent for all blood sample analyses, and the protocol was approved by the Ethical Committee of Kyoto University Graduate School of Medicine. Sample analyses were also conducted in accordance with the policies and procedures of the Institutional Review Board for the use of human subjects in research at the Diagnostics Division of Shionogi & Co., Ltd.

### Peptides and Reagents

Glycosylated proBNP and recombinant proBNP were purchased from Hytest Ltd. (Turk, Finland). The protein content was determined by amino acid analysis. BNP was from Peptide Institute, Inc. (Osaka, Japan). EZ-Link-sulfo-NHS-biotinylation kits were from Pierce (Rockford, IL). Sulfo-HMCS (N-(8-maleimidocapryloxy) sulfosuccinimide) was from Dojindo (Kumamoto, Japan). CDP/E (Disodium 2-chloro-5-(4-methoxy-spiro{1,2-dioxetane-3,2′-(5′-chloro)-tricyclo [3,3,1,13,7]decan}-4-yl)-1-phenyl phosphate) was from Applied Biosystems (Foster City, CA).

### Antibodies

The monoclonal antibodies BC203 (IgG1, k) and KY-BNP-II (IgG1, k) were developed by Shionogi & Co., Ltd [10]. BC203 and KY-BNP-II recognize the C-terminal region and the ring region of BNP, respectively. The monoclonal antibody 18H5 was purchased from Hytest Ltd. 18H5 recognizes a region (a.a. 13–20) of proBNP. In the proBNP assay, the combination of BC203 (capture) and 18H5 (detection) was used because 18H5 is not affected by glycosylation [11]. In the total BNP assay, the combination of BC203 (capture) and KY-BNP-II (detection) was used because KY-BNP-II recognizes nearly all bioactive BNPs (Figure 1).

### Preparation of BC203 coated immunoassay plates

BC203, which was the capture antibody in both assays, was biotinylated using an EZ-Link-sulfo-NHS-biotinylation kit according to the manufacturer's instructions. The biotinylated BC203 (0.2 mg/well in 100 mL PBS) was added to streptavidin-coated plates and incubated for 18 h at 4°C. After washing with a saline containing 0.01 g/dL Tween 20 and 0.05 g/dL sodium azide (Wash Buffer), the BC203 coated immunoassay plates were dried in a desiccator.

### Preparation of 18H5 (Fab')-ALP and KY-BNP-II (Fab')-ALP

The 18H5 and KY-BNP-II mAbs (IgG) were digested with pepsin (IgG/pepsin = 1/0.05) for 4 h at 37°C in 100 mM citrate buffer (pH 4.0) containing 100 mM NaCl. Thereafter, Fab' solution was prepared by reduction with 10 mM 2-mercaptoethylamine in 0.1 M phosphate buffer (pH 6.0) containing 5 mM EDTA using the standard method [12]. Alkaline phosphatase from calf intestine (ALP; 2.0 mg or 14.2 nmol; Kikkoman, Chiba, Japan) in 0.475 mL 0.1 M Tris-HCl buffer (pH 7.0) containing 1 mM MgCl2 and 0.1 mM ZnCl2 was mixed with 31 mg (71 nmol) of Sulfo-HMCS in 0.05 mL of water for 1.5 h on ice, after which the HMCS-activated ALP was purified on a PD-10 column (GE Healthcare, Chalfont St. Giles, UK). Aliquots of HMCS-activated ALP solution (0.96 mg in 0.192 mL) were each added to 0.441 mg of the Fab' in 0.15 mL of 0.1 M phosphate buffer (pH 6.0) containing 5 mM EDTA and mixed for 16 h at 4°C. Unlabeled Fab' antibody was removed using a TSKgel 3000SWxl column. The purified 18H5 (Fab')-ALP and KY-BNP-II (Fab')-ALP were then diluted with a StabilZyme AP (BioFX Lab.) and stored at 4°C until use.

### Sandwich 2-step Chemiluminescent Enzyme Immunoassay

After the BC203 coated immunoassay plates were washed with a wash buffer, 50 mL of test sample or calibrator and 50 mL of Assay Buffer (0.05 M Tris-HCl buffer (pH 7.4), 1 g/dL BSA, 0.01 g/dL Tween80, 1 mM MgCl2, 0.1 mM ZnCl2, 1000K IU/mL Aprotinin, 0.1 mg/mL mouse gamma globulin, 0.9 g/dL NaCl) were added to the wells. The plates were then incubated for 3 h at 25°C. After washing with wash buffer, 100 mL of detection antibodies (18H5 (Fab')-ALP, 100 ng/ml; KY-BNP-II (Fab')-ALP, 416 ng/ml) were added to the wells. The plates were then incubated for 1 h at 25°C, followed by washing with wash buffer and addition of substrate (CDP/E) solution. The chemiluminescence from each well was then measured using a plate reader (Wallac 1420 Arvo sx, Perkin Elmer, Inc., MA).

### Study Patients

We collected blood samples from heart failure patients (18 men and 14 women; age range, 34–84 years, mean age, 65±11 years) hospitalized at Kyoto University Hospital. The primary causes of the heart failure were ischemic heart disease (n = 8), cardiomyopathy (n = 8), valvular heart disease (n = 7), pulmonary hypertension (n = 7) and others (n = 2), which were diagnosed from the medical history, physical examination and chest radiographic, electrocardiographic, echocardiographic and/or cardiac catheterization findings. Patients with symptomatic heart failure were under medication, including angiotensin-converting-enzyme inhibitors/angiotensin-receptor blockers, digitalis and diuretics. The New York Heart Association (NYHA) functional classes were class I–II (n = 19) and class III–IV (n = 13). Healthy subjects (61 men and 54 women; age range, 30–78 years, mean age, 50±10 years) were selected based on their normal physical, laboratory, chest radiographic, electrocardiographic and echocardiographic findings, and their BNP levels.

### Plasma samples

Blood samples were drawn into plastic syringes and quickly transferred to chilled tubes containing EDTA (1.5 mg/mL, blood) and aprotinin (500 U/mL blood) and centrifuged at 1600× g for 20 min at 4°C. The obtained plasma samples were stored at −80°C until assayed.

### Assay of plasma NT-proBNP levels

Plasma levels of NT-proBNP were measured using Elecsys proBNP II assay system (Roche Diagnostics, Basel, Switzerland).

### Gel filtration chromatography

Plasma samples were extracted using Sep-Pak C18 cartridges (Waters, Milford, MA, USA) as previously described [6]. The eluate was lyophilized and dissolved in 30% acetonitrile containing 0.1% TFA. The resultant solution (300 ml) was separated by gel filtration HPLC on a Superdex 75 10/300 GL columns (10×300 mm×2, GE Healthcare) in the same buffer at a flow rate of 0.4 mL/min. The column effluent was fractionated every minute into polypropylene tubes containing bovine serum albumin (100 mg) and each fraction was analyzed using the total BNP and proBNP assay systems. Because recent studies have shown that glycosylated proBNP with a MW of about 30 K circulates in the plasma [7], we examined the gel filtration positions at which commercial recombinant proBNP and glycosylated proBNP, and synthetic BNP were eluted to determine which is the major molecular form of BNP in human plasma.

### Deglycosylation enzyme treatment

We further analyzed the immunoreactive proBNP levels to determine whether immunoreactive proBNP in plasma is glycosylated. Eluate lyophilized after extraction on a Sep-Pak C18 column was dissolved in phosphate buffer and incubated with or without a cocktail of deglycosylation enzymes for 24 h at 37°C, as previously described [13]. The enzyme cocktail included O-glycosidase (Roche Diagnostic) and neuraminidase (Roche Diagnostics) at final concentrations of 4.25 and 42.5 mU/mL, respectively. These two enzymes were essential for the deglycosylation, and the enzyme concentrations and incubation period were selected based on the results of preliminary and previously reported studies [11], [13], [14]. We then lyophilized the sample again and dissolved it in 30% acetonitrile containing 0.1% TFA, after which it was subjected to gel-filtration HPLC as described above.

### Statistical Analysis

All values are expressed as means ± SD. The statistical significance of differences between 2 groups was evaluated using Fisher's exact test or unpaired Student's t test, as appropriate. Variables were compared among three groups using one-way analysis of variance followed by Bonferroni's multiple comparison test. Correlation coefficients were calculated using linear regression analysis. Values of P<0.05 were considered significant.

## Results

### Standard curve, recovery and precision

**Figure 2:**
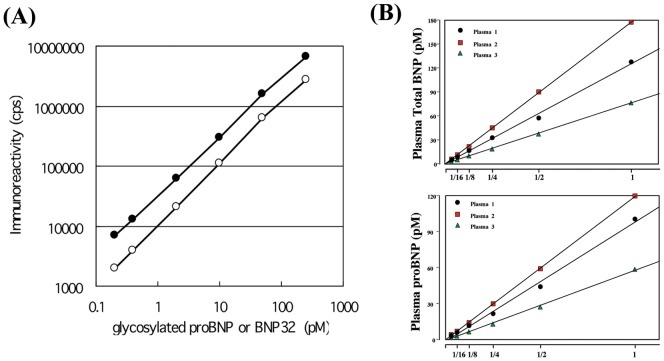
Standard curves for the proBNP (open circle) and total BNP (closed circle) assays (A). Plasma dilution curves (B). Three plasma samples collected from three heart failure patients were serially diluted with buffer.

Figure 2 shows typical standard curves for the proBNP and total BNP assay systems. The lower detection limits were 0.04 pmol/L (proBNP) and 0.02 pmol/L (total BNP). At these levels the mean value (n = 8 each) of the chemiluminescence intensity (cps) was more than twice that at 0 pmol/L (P<0.05). The working range (coefficient of variation (CV)<15%) of both assays was 0.2–250 pmol/L in total BNP and 0.4–250 pmol/L in proBNP, respectively.

Table 1 shows the recovery of standard proBNP and BNP, which was estimated from the levels of glycosylated proBNP or BNP added to clinically available plasma (endogenous total BNP = 0.3 pmol/L and proBNP = 0.2 pmol/L). In the proBNP assay system, using glycosylated proBNP as a standard, the recovery ranged from 90–101%. In the total BNP assay system, using BNP as the standard the recovery ranged from 85–97%. The effect of diluting plasma samples containing 100 pmol/L glycosylated proBNP or BNP is shown in Table 2. At every dilution level, the recovery rate was good. We also investigated the effects of dilution on plasma levels of total BNP and proBNP in three heart failure patients. As shown in Figure 2B, the calculated total BNP and proBNP values varied linearly with dilution (correlation coefficients = 0.998–1.00).

**Table 1:** Recovery of standard glycosylated proBNP and BNP added to human plasma.

Added peptide concentration, pmol/L	Recovery, %	Recovery, %
	proBNP assay system	total BNP assay system
2.0	90	85
25.0	101	97
100.0	95	95

**Table 2:** Effects of dilution on recovery rates with the proBNP and total BNP assay systems.

Dilution magnitude	proBNP assay system		total BNP assay system	
	Measured, pmol/L	Recovery, %	Measured, pmol/L	Recovery, %
1	94	-	95	-
2	105	112	101	107
5	96	102	104	109
10	92	98	92	97
20	97	103	93	98
50	99	105	97	103
100	87	92	95	100

When we then assessed the intra- and inter-assay precision using plasma spiked with glycosylated proBNP or BNP, we found that the intra-assay CV ranged from 5.2%–8.0% in proBNP assay and from 7.0%–8.4% in total BNP assay, while inter-assay CV ranged from 5.3–7.4% in proBNP assay and from 1.9%–9.5% in total BNP assay, respectively (Table 3, 4).

**Table 3:** Intra- and Inter-assay precision of the proBNP assay systems.

	Added proBNP concentration pmol/L	Measured concentration pmol/L		CV	Bias
		Mean	S.D.	%	%
Intra-assay (n = 5)	2.0	2.0	0.2	8.0	2.0
	25	25	1.3	5.2	0.0
	100	101	5.5	5.4	1.0
Inter-assay (n = 15)	2.0	1.9	0.1	5.3	−5.8
	25	23	1.7	7.4	−8.0
	100	96	6.1	6.4	−4.0

**Table 4:** Intra- and Inter-assay precision of the total BNP systems.

	Added BNP concentration pmol/L	Measured concentration pmol/L		CV	Bias
		Mean	S.D.	%	%
Intra-assay (n = 5)	2.0	2.3	0.2	7.0	15.0
	25	25	2.1	8.4	1.0
	100	99	7.1	7.2	−0.7
Inter-assay (n = 15)	2.0	2.1	0.2	9.5	5.0
	25	24	1.7	2.9	−4.0
	100	100	1.9	1.9	0.0

### Specificity and sensitivity

We next examined the cross-reactivity between proBNP and BNP. As shown in Table 5, the presence of BNP did not affect the values measured with the proBNP assay system. Moreover, the values measured with the total BNP assay system were the sum of the BNP and proBNP even at different compositions of these two peptides. Thus, the total BNP assay recognized both BNP and proBNP with the same efficiency and sensitivity. Likewise, the proBNP and total BNP assay systems recognized proBNP with the same efficiency and sensitivity.

**Table 5:** Cross-reactivity between proBNP and BNP.

Added peptide concentration, pmol/L	Added peptide concentration, pmol/L	Measured peptide concentration, pmol/L	Measured peptide concentration, pmol/L
proBNP	BNP	proBNP assay	total BNP assay
50	50	58	114
100	10	113	119
10	100	8	113

### Gel-filtration chromatography before and after deglycosylation procedure

**Figure 3:**
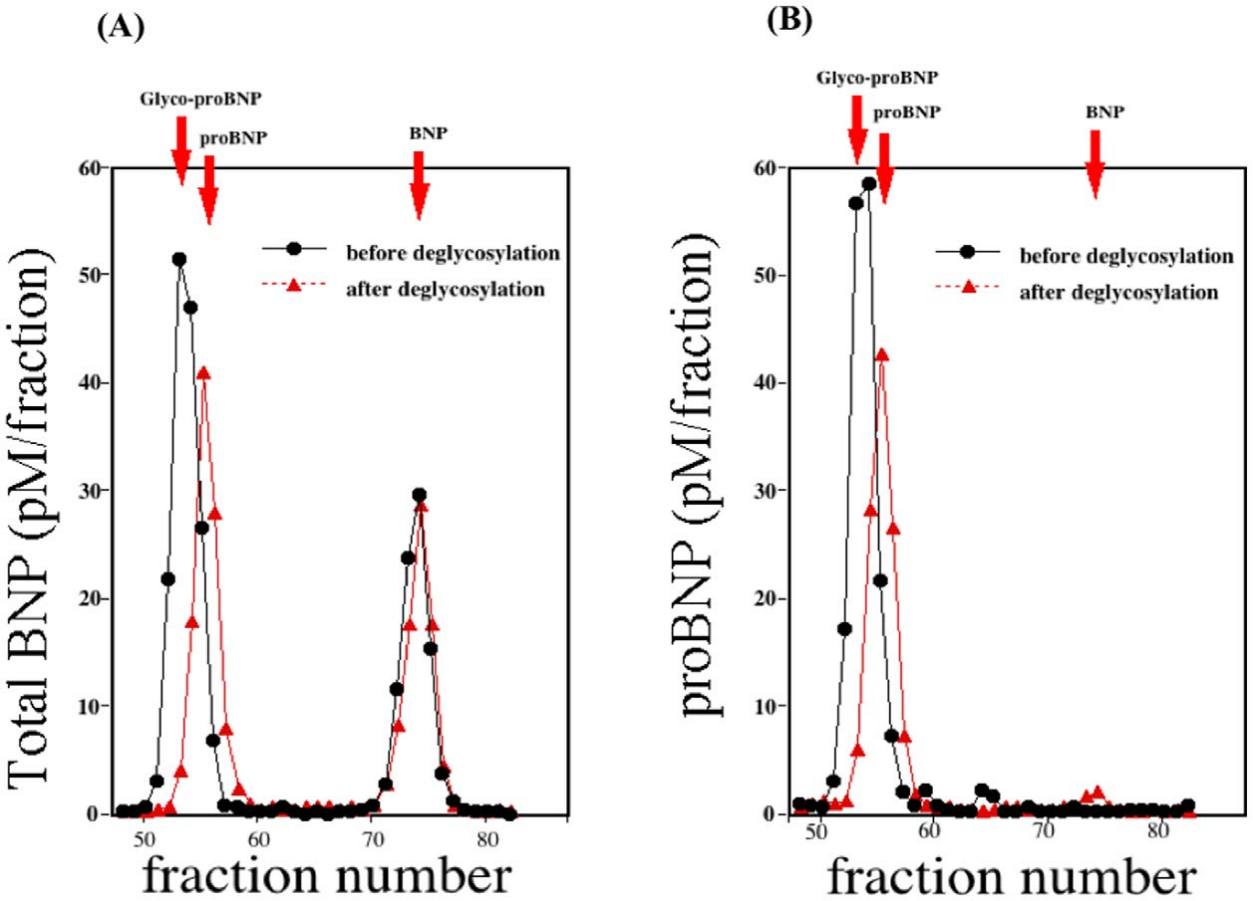
Gel filtration analysis of total BNP (A) and proBNP (B) in plasma from a heart failure patient. Fractions were assayed using the total BNP (A) and proBNP (B) systems. The elution points for glycosylated proBNP, proBNP and BNP are indicated by red arrows. Black and red lines respectively show gel filtration analyses of total BNP (A) and proBNP (B) in the same plasma sample before and after deglycosylation.

Figure 3-A shows two immunoreactive BNP peaks detected using the total BNP assay with HPLC fractions. The first peak appeared in fractions 52–55 and the second peak in fractions 72–75. With the same sample, one immunoreactive BNP peak was detected by the proBNP assay (Figure 3-B); the position of that peak was completely consistent with the proBNP peak obtained with the total BNP assay. When subjected to gel filtration HPLC, recombinant proBNP, glycosylated proBNP and BNP were eluted mainly in fractions 53, 56 and 74, respectively. Treating the same plasma sample with an enzyme cocktail catalyzing deglycosylation shifted the first peak to fraction 54–56, which is consistent with the proBNP peak. From these results, we conclude that total BNP assay evaluates the sum of the glycosylated proBNP plus BNP, while proBNP assay detects glycosylated proBNP. The proBNP was not detected in a significant level with either assay system.

### Plasma concentrations of proBNP, total BNP, and NT-proBNP in healthy subjects and heart failure patients

**Figure 4:**
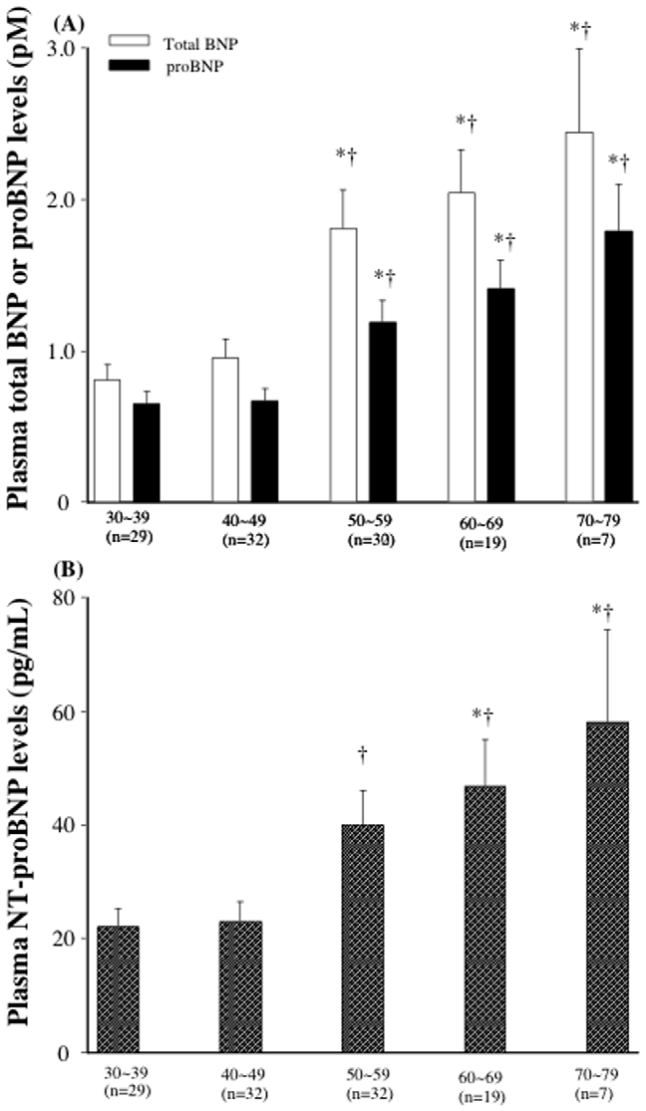
Plasma Levels of total BNP, proBNP, and NT-proBNP in different age groups. Bar graph showing the total BNP, proBNP (A) and NT-proBNP levels (B). Values are means ± SE., *P<0.05 vs total BNP, proBNP, and NT-proBNP in 30∼39, †P<0.05 vs total BNP, proBNP, and NT-proBNP in 40∼49.

**Figure 5:**
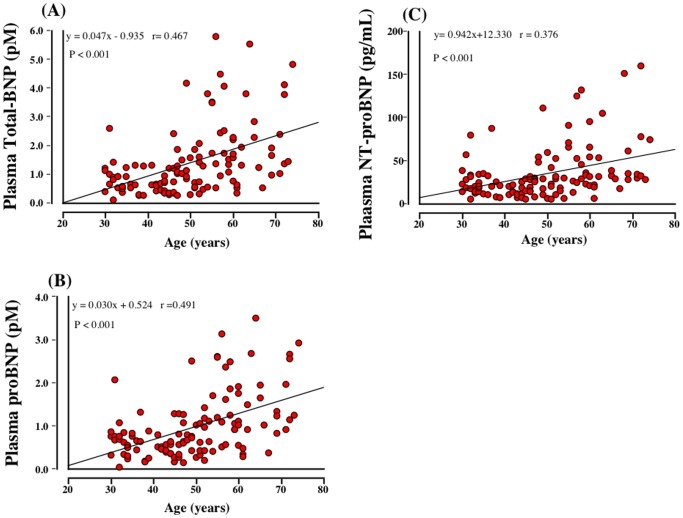
The relationships between total BNP (A), proBNP (B), and NT-proBNP (C) and age.

Plasma total BNP, proBNP and NT-proBNP levels in different age groups were shown in Figure 4-A, B. Plasma total BNP, proBNP and NT-proBNP levels appeared to increase according to the age. The older age groups (more than 50) had higher total BNP, proBNP and NT-proBNP levels than younger age groups (less than 50); however, there were no statistical differences in NT-proBNP between 30∼39 and 50∼59. In addition, there were significant positive relationships between plasma total BNP (r = 0.467, p<0.001), proBNP (r = 0.491, p<0.001) and NT-proBNP (r = 0.376, p<0.001) levels and age (Figure 5-A, B, C).

**Figure 6:**
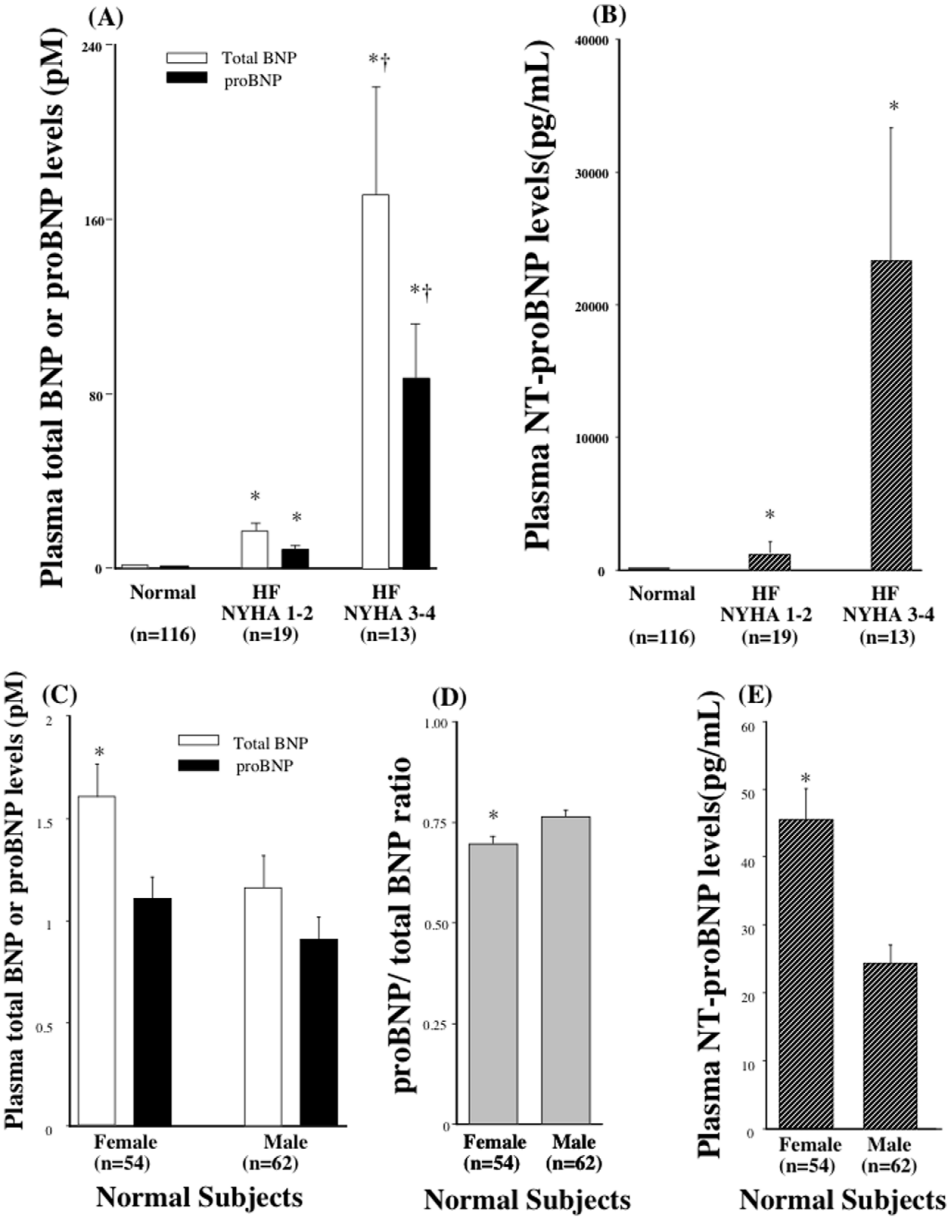
Plasma Levels of proBNP, total BNP, and NT-proBNP in normal and heart failure. Bar graph showing the total BNP, proBNP (A) and NT-proBNP (B) levels in healthy subjects and heart failure patients with NYHA classes 1–2 and 3–4. *P<0.05 vs total BNP and proBNP in normal, †P<0.05 vs total BNP and proBNP in HF NYHA 1–2. Bar graph showing the total BNP, proBNP (C), proBNP/total BNP ratio (D) and NT-proBNP (E) levels in male and female in healthy subjects. Values are means ± SE. *P<0.05 vs male.

The mean total BNP and proBNP in plasma from 116 healthy subjects were 1.4±1.2 pM and 1.0±0.7 pM, respectively (Figure 6-A). Female had higher total BNP than male (total BNP: 1.7±1.3 vs 1.1±1.1, P<0.05; proBNP: 1.1±0.8 vs 0.8±0.6 pM, P = 0.11) (Figure 6-C). proBNP/total BNP ratio was lower in female than that in male. NT-proBNP was also higher in female than those in male (Figure 6-E). The total BNP and proBNP levels were markedly elevated in heart failure patients, and the magnitude of the increase reflected the severity of the patients' condition as observed in NT-proBNP (Figure 6-A, B).

## Discussion

Plasma levels of the cardiac hormone BNP increase in proportion to the severity of heart failure. Indeed, plasma BNP levels are used as a biomarker of heart failure, and the guidelines in many countries recommend that BNP be used as a diagnostic indicator of acute and chronic heart failure [1]–[3]. The stimuli that increase cardiac BNP production include pressure overload, volume overload and ischemia, as well as various cytokines and neurohumoral factors [15]. In response to these stimuli, BNP mRNA expression is rapidly upregulated. Following translation of the protein, the signal peptide is removed to produce proBNP, which is then cleaved into BNP and the NT-proBNP fragment during secretion [15]. It is noteworthy that BNP and proBNP could not be distinguished from one another in earlier BNP assay systems because the anti-BNP antibodies cross-reacted with proBNP. We therefore endeavored to develop a new assay system that would enable separate measurement of BNP and proBNP. Recent studies have shown that levels of uncleaved proBNP are increased in heart failure to a greater degree than BNP [5]–[7], [16]. Using a combination of gel filtration and an immunoenzyme fluorescent assay for BNP, we previously found that proBNP levels are increased in heart failure and that the proBNP/total BNP ratios are higher in heart failure patients with ventricular overload than those with atrial overload [6]. Although this protocol provides useful information, the methodology is time-consuming and impractical for routine assays in clinical laboratories. In addition, recovery of proBNP may be diminished by both extraction and the gel filtration steps [9], [16]. To overcome these problems, we developed new direct immunochemiluminescent assays for proBNP and total BNP.

We used two monoclonal antibodies, BC203 and 18H5, to assay proBNP. BC203 recognizes an epitope in the C-terminal of proBNP, while 18H5 recognizes an epitope in the N-terminal. Recent studies showed that proBNP has seven sites suitable for *O*-linked oligosaccharide attachment (Ser36, Thr37, Thr44, Thr48, Thr53, Ser58 and Thr71) within the N-terminal portion of the peptide [14]. Because the *O*-linked oligosaccharide attachments almost completely inhibit the binding of the antibody to the peptide [17], we selected 18H5, which recognizes the N-terminal of proBNP (a.a. 13–20) in a region not subject to glycosylation (Figure 1). To assay total BNP, we used the monoclonal antibodies BC203 and KY-BNP-II, as previously reported [10]. In both assays, BC203 served as the capture antibody. Importantly, because the affinity of 18H5 for the N-terminal portion is similar to the affinity of KY-BNP-II for the ring structure, we are able to calculate the proBNP/total BNP ratio. In addition, our new assays are less time-consuming and more sensitive and accurate than earlier ones, and the lower detection limits for total BNP (0.02 pmol/L) and proBNP (0.04 pmol/L) enabled us to measure plasma proBNP levels in nearly all the healthy subjects tested.

We used gel-filtration on two tandemly connected Superdex 75 columns to determine the molecular mass of plasma proBNP. As shown in Figure 3-A,B, a single peak of proBNP was obtained in both the total BNP and proBNP assay systems. The elution points are consistent with that of glycosylated proBNP, but not deglycosylated proBNP, and deglycosylation treatment significantly shifted the peak rightward (Figure 3-A,B) to an elution point consistent with proBNP. The peak immunoreactivity of proBNP after deglycosylation was slightly smaller than before treatment, suggesting the recovery rate of proBNP after gel-filtration is lower than that of glycosylated proBNP, which is consistent with proBNP being more adsorptive than glycosylated proBNP. Our findings are also consistent with previous Western blot analyses showing that plasma levels of glycosylated proBNP are elevated and no substantial level of proBNP is detected in severe heart failure [7]. Taken together, these results suggest that the major molecular form of proBNP in the plasma of patients with heart failure is the glycosylated form.

ProBNP is also the important molecular form of BNP in the plasma of healthy subjects. When we previously used gel-filtration and a fluorescent immunoenzyme assay to measure BNP and proBNP, we found that levels of BNP were slightly higher than those of proBNP in both healthy subjects and heart failure patients. The exact reason for the discrepancy in proBNP levels between the earlier study and the present one is unclear; however, the lower recovery caused by the need for extraction from plasma on a Sep-Pak C18 cartridge may have contributed to the lower proBNP levels in the earlier study [9], [16]. Recent studies have shown that proBNP has much less ability to induce cGMP production in vascular smooth muscle and endothelial cells than BNP [7], [18]. This suggests that increases in the levels of the low-activity proBNP in heart failure may contribute to the so-called “BNP paradox” [19]. That is, administration of exogenous recombinant human BNP to heart failure patients has a substantial clinical and hemodynamic impact, despite the presence of high levels of immunoreactive BNP in their plasma, as measured with commercially used BNP assays.

In the current study, we showed that total BNP and NT-proBNP increased with aging, which are consistent with the previous studies. In addition, the current study first showed that plasma proBNP level increased with aging. However, there were no statistical differences in NT-proBNP between 30∼39 and 50∼59, whereas there were significant differences in total and proBNP between 30∼39 and 50∼59, suggesting that total and proBNP are more sensitive than NT-proBNP. In addition, total and proBNP seemed to be well correlated with age (r = 0.467. 0.491, each) than NT-proBNP (r = 0.376). Thus, total BNP and proBNP may be better marker in discriminating the effect of age than NT-proBNP. Increased myocardial mass and/or reduction of renal clearance of natriuretic peptides with aging may be one of the possible reason for increased BNP and NT-BNP with aging; however, exact mechanism for it still remains unknown and further study is necessary to investigate the relationships between proBNP and aging.

We also analyzed the effects of gender on proBNP, total BNP, proBNP/total BNP ratio and NT-proBNP. Interestingly, in female higher total BNP and NT-proBNP and lower proBNP/total BNP ratio without changing of proBNP was observed. Calculated BNP (total BNP - proBNP) was also increased. This finding may be explained that increased proBNP production and higher processing rate. Further study is necessary to elucidate the mechanism of increased total BNP and NT-proBNP and lower proBNP/total BNP ratio without changing of proBNP in female.

In summary, we have developed rapid and precise immunochemiluminescent assay systems for routine determination of total BNP and proBNP levels in human plasma. Using these assay systems we showed that in addition to BNP, considerable amount of proBNP circulates in both healthy subjects and heart failure patients. The precise relation of the proBNP/total BNP ratio to heart failure remains unknown, as does whether the heart mainly secretes proBNP. Likewise, the effects of age, sex and renal function on proBNP levels remain unknown. We anticipate our new assays for the proBNP and total BNP will be helpful for addressing each of those issues.
